# The Impact of 120 Minutes of Match-Play on Recovery and Subsequent Match Performance: A Case Report in Professional Soccer Players

**DOI:** 10.3390/sports6010022

**Published:** 2018-03-13

**Authors:** Nathan Winder, Mark Russell, Robert J. Naughton, Liam D. Harper

**Affiliations:** 1 Leeds United Football Club, Leeds LS23 7BA, UK; nathan.winder@leedsunited.com; 2 School of Social and Health Sciences, Leeds Trinity University, Leeds LS18 5HD, UK; m.russell@leedstrinity.ac.uk; 3 School of Human and Health Sciences, University of Huddersfield, Huddersfield HD1 3DH, UK; r.naughton@hud.ac.uk

**Keywords:** football, accelerometry, fatigue

## Abstract

The influence of a match including extra-time (ET) on subsequent 90 min match performance and recovery has not been investigated. Four professional soccer players played in three competitive matches in a 7-day period: matches one (MD1) and three (MD3) lasted 90 min and match 2 (MD2) lasted 120 min (i.e., included ET). Physical (total and high-intensity (HI) distance covered, accelerations and decelerations, and mechanical load) and technical performances (pass and dribble accuracy) were analyzed throughout match-play. Subjective measures of recovery and countermovement jump (CMJ) height were made 36–42 h post-match. Post-MD2, there were very or most likely harmful effects of ET on CMJ height (−6 ± 9%), muscle soreness (+18 ± 12%), and fatigue (+27 ± 4%) scores, and overall wellness score (−13 ± 5%) compared to post-MD1. Furthermore, there were very likely harmful effects on muscle soreness (+13 ± 14%), wellness scores (−8 ± 10%), and CMJ height (−6 ± 9%) post-MD3 vs. post-MD1. There was a possibly harmful effect of ET on HI distance covered during MD3, along with reductions in pass (−9.3%) and dribble (−12.4%) accuracy. An ET match negatively impacted recovery 36 h post-match. Furthermore, in some players, indices of performance in a 90 min match played 64 h following ET were compromised, with subsequent recovery also adversely affected.

## 1. Introduction

The influence of 120 min of soccer match-play (i.e., matches requiring a 30 min extra-time period; ET), on physical and technical performance, and functional and biochemical measures of recovery has recently been investigated [1,2,3,4]. High-intensity distance covered and distance covered per minute reduces during the two 15 min periods of ET compared to other 15 min periods during competitive soccer match-play [3]. Furthermore, the number of passes and dribbles reduces during the last 15 min of ET compared to prior 15 min periods played during elite professional soccer matches [1]. Neuromuscular fatigue of central and peripheral origin develops during ET, which negatively impacts sprint performance and muscle force production [5], with reductions in muscle glycogen and an increased reliance on fat as a fuel contributing to this fatigue response [4]. Moreover, reductions in countermovement jump (CMJ) height and elevations in creatine kinase concentrations persisted 48 h after an English Premier League Reserve match requiring ET [3]. However, the effect of 120 min match-play on subjective measures of post-match recovery and subsequent performance during a 90 min competitive match requires investigation.

Soccer players can often be exposed to congested fixture schedules, which can influence performance and injury risk [6,7,8]. Despite rotation of squads preventing some players from exposure to congested schedules, ~40% of players complete all matches during a two or three 90 min match microcycle [7]. As transient reductions in performance have been observed during both 90 and 120 min matches, it is important to be able to assess measures of post-match recovery and subsequent performance during successive matches, particularly during periods of fixture congestion.

The aim of this study was twofold: (1) use GPS-derived data to assess the impact of playing a competitive match requiring ET on a competitive 90 min match played 64 h later; (2) elucidate the influence of ET on post-match recovery.

## 2. Materials and Methods

Four male players (two center backs, one full back, one center midfielder; age: 24 ± 3 years; body mass: 85.6 ± 6.3 kg; body fat: 9.1 ± 0.4% (as measured by skinfold calipers); stature: 1.89 ± 0.4 m; *V*O_2max_: 61.0 ± 1.8 mL·kg·min^−1^) from a club playing in the third tier of English professional soccer (now in the second tier) gave informed consent to participate in this investigation. All players agreed for their data to be used for research purposes and as part of internal club procedures regarding performance and recovery assessment via written consent.

**Figure 1:**
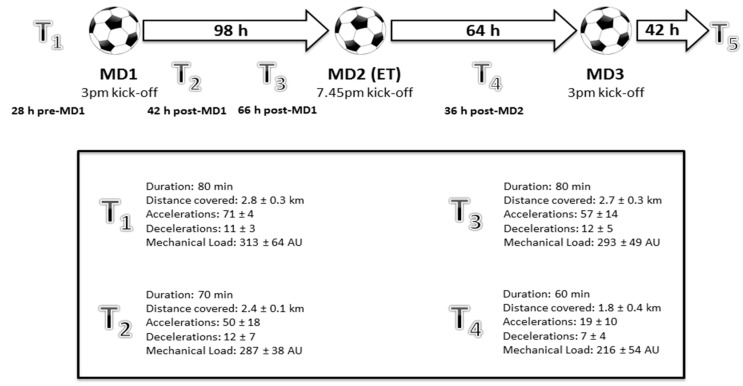
Schematic illustrating the study design. MD = match-day. T = training. Thresholds for accelerations and decelerations are >2 m·s^−2^ and <−2 m·s^−2^, respectively. Mechanical load derived from the manufacturer (PlayerLoad^TM^; Catapult Innovations, Melbourne, Australia).

All players participated in three competitive matches in a seven-day period, with recovery measures taken 36–42 h post-match, prior to team training (Figure 1). Both match-days one (MD1) and three (MD3) were 90 min league matches played against opposition in the same tier, with MD1 played at the study team’s home venue and MD3 played at the opposition’s venue. Match-day two (MD2) was a cup match that had a duration of 120 min (i.e., included ET) played at the study team’s home venue vs. a team from the first tier of English football. Players wore 10 Hz GPS units (Catapult MinimaxX V4, Catapult Innovations, Melbourne, Australia) during matches and training which were used to measure (presented as absolute values and per min^−1^): total and high-intensity distance covered (>18 km·h^−1^), number of accelerations (>2 m·s^−2^) and decelerations (<−2 m·s^−2^), and an indicator of mechanical load derived from the manufacturer (PlayerLoad^TM^; Catapult Innovations, Melbourne, Australia). The PL_Total_ (overall mechanical load) metric has previously been shown to possess good convergent validity with measures of exercise intensity and good test-retest reliability [9], as well as good within- and between-device reliability [10]. Furthermore, the accuracy of the MinimaxX V4 for measurement of distances covered at high speeds has been demonstrated [11]. All matches were filmed, and video footage was subsequently analyzed using a notational analysis procedure (similar to the methods of Harper et al. [1]) to assess pass and dribble accuracy (for operational definitions, see [1]).

Recovery measures included subjective measures of mood, fatigue, sleep, and muscle soreness (rated by players on a 1–7 scale (1 = best, 7 = worst) using a bespoke smartphone app), which were also used to create a composite wellness score (sum of four scores divided by 28, multiplied by 100). Countermovement jump (performed with hands on hips) height was measured using an electronic mat (Just Jump System, Perform Better Ltd., Warwickshire, UK).

### Statistical Analysis

Data are presented as mean ± standard deviation (SD). Magnitude-based inferences regarding the effect of ET on recovery and performance were derived utilizing methods described previously using a published spreadsheet [12]. Smallest worthwhile change was defined for each variable as 0.2 of the between-player SD. Clinical inference criteria were used to classify the effects of ET, with likelihoods classified as: most unlikely (0–5%), very unlikely (0.5–5%), unlikely (5–25%), possibly likely (25–75%), likely (75–95%), very likely (95–99%), most likely (>99%). As pass and dribble accuracy were measured as a cumulative total of all four players, between-match statistical analysis was not possible. Effect sizes (ESs) were calculated using Cohen’s *d* for parametric data and Z distribution values for non-parametric data, with thresholds of 0.2, 0.5, and 0.8 considered as small, medium, and large ESs, respectively [13]. 

## 3. Results

Descriptive match performance and recovery data are presented in Table 1 and Table 2 along with mean differences (±CL; confidence limits), qualitative inferences, and ESs.

### 3.1. Influence of ET on Subsequent 90 Min Performance

There was a possibly harmful effect of ET on HI distance covered during MD3 (Table 2). However, unclear effects were found for all other physical performance variables, although there were medium to large ESs for decrements in distance covered, and elevations in mechanical load (Table 2). Additionally, both pass and dribble accuracy were respectively 9.3% and 12.4% lower during MD3 vs. MD1 (Table 2).

### 3.2. Influence of ET on Recovery

Notably, post-MD2, there were very or most likely harmful effects of ET on CMJ height (−6 ± 9%), muscle soreness (+18 ± 12%), and fatigue (+27 ± 4%) scores, and overall wellness score (−13 ± 5%) compared to post-MD1 (Table 1). Nonetheless, mood and sleep scores remained unchanged (Table 1). Furthermore, we identified very likely harmful effects on muscle soreness (+13 ± 14%), wellness scores (−8 ± 10%), and CMJ height (−6 ± 9%) post-MD3 vs. post-MD1, with no differences in mood, fatigue, or sleep (Table 2).

### 3.3. Individual Results

All player’s wellness scores reduced following MD2 vs. MD1 (Table 3), with each sub-variable changing negatively (i.e., increasing) or remaining the same, except for sleep for Player 2. Countermovement jump height reduced in three out of the four players (although this was trivial for Player 2). Relative to match duration, there was an increase in performance output for all four players for each measured variable (distance covered, HI distance covered, number of accelerations and decelerations, and mechanical load) in MD2 vs. MD1, except for number of accelerations for Player 2 (Table 3). 

The HI distance covered values for two players reduced from an average of 6.6 to 4.4 m·min^−1^ and 4.87 to 3.54 m·min^−1^ during MD1 vs. MD3, respectively, despite both maintaining a similar total distance covered (120 vs. 119 and 108 vs. 110 m·min^−1^, respectively). However, the HI distance covered for the other two players actually increased from an average of 3.3 to 4.0 m·min^−1^ and 4.7 to 5.0 m·min^−1^ with similar distances covered (109 vs. 114 and 122 vs. 120 m·min^−1^, respectively) during MD1 vs. MD3 (Table 4). The players who had a reduction in HI distance covered during MD3 were not exposed to higher numbers of accelerations or decelerations or a higher mechanical load during MD2 than the other players, and neither player’s change in subjective recovery or CMJ height was markedly different to the two other players.

## 4. Discussion

Aligning with our aims, we have demonstrated that there is compromised recovery 48 h following a match requiring ET in some individual players. Furthermore, performance and recovery were potentially exacerbated during, and following, a match played 64 h later.

We observed that players cover ~5 km more distance, ~400 m more HI distance, and perform an additional ~170 accelerations and ~90 decelerations during ET vs. a 90 min match (Table 1). These findings are similar to Russell et al., who used a within-match approach to analyze temporal GPS data during an English Premier League reserve cup match [3]. As ET seems to create a larger load than that observed during 90 min, the impact of ET should be accounted for in the training and recovery of players.

We observed perturbations in ratings of fatigue, muscle soreness, overall wellness scores, and CMJ height 36 h following a match requiring ET (MD2; Table 1 and Table 3). Therefore, it would seem ET has harmful effects on indices of recovery, as Russell et al. also observed decrements in CMJ performance 24 and 48 h following a match requiring ET, as well as elevations in concentrations of creatine kinase [3]. We observed very likely harmful effects on muscle soreness and wellness scores, and CMJ height 48 h post-match when comparing MD3 to MD1, including medium to large ESs for all measured recovery variables except sleep, in some of the players (Table 2 and Table 4). This may indicate that recovery is exacerbated due to the extended duration of MD2. However, as shown in Table 1 and Table 3, the relative intensity of MD2 was higher than MD1 (i.e., total and HI distance covered per min, the number of accelerations and decelerations per min, and mechanical load per min were all higher in MD2 vs. MD1). As such, this may have contributed to the depressed recovery response compared to MD1, rather than just match duration per se. Furthermore, the fact that the matches were played in a short period of time (i.e., congested fixture schedule) may have influenced the recovery response following MD3. Nonetheless, little data exists profiling players’ perceptual recovery responses during periods of fixture congestion when all matches are 90 min, making comparisons difficult. 

We also observed a possibly harmful effect on HI distance covered during MD3; however, the effect on all other physical performance variables was unclear, with a medium ES for reductions in distance covered. At an individual level, this was particularly apparent in two of the four players. However, there was no particular variable measured during and after MD2 that may explain this change in performance in these two players. A number of factors may have contributed to this difference, including the quality of opposition faced directly in that position (i.e., central defender vs. opposition striker) or the tactics of the opposition team as a whole. Additionally, both pass and dribble accuracy were reduced during MD3 vs. MD1 (Table 2). Therefore, playing in a match requiring ET may have implications for both physical and technical performance in a subsequent 90 min match of close temporal proximity. Although further research is required to delineate the influence of fixture congestion per se vs. the knock-on effect of ET, no previous data has shown that fixture congestion of a similar nature (i.e., three matches in seven days) causes reductions in physical or technical performance [7]; however, we acknowledge the inferential limitations of our small dataset and sample size.

The differences in performance and recovery parameters between matches may be due to a number of factors. Firstly, readers should be cognizant of the small sample size (four professional players), and the effect this may have had on the statistical analyses of group data (i.e., inherent variation between individuals influencing the statistical outcome). Therefore, we also reported individual data for each player. The opposition in MD2 played in the first tier of English football (47 league places difference at the time of the match). We also acknowledge the influence of extraneous factors such as score-line and self-pacing strategies [14]. Moreover, MD1 was played at home and MD3 away (80 miles round trip); however, team tactics and formation were similar between matches. Furthermore, singular match-to-match comparisons can be difficult due to inherent match-play variability [15]; nonetheless, the performance and recovery data from MD1 is analogous with a ~4-match average in the same group of players. There is a need for robust investigations with large datasets from multiple professional clubs, or the use of controlled soccer-specific protocols (i.e., [4]) with an array of recovery measures (i.e., physiological and biochemical) to further enhance knowledge of this topic.

From a practical perspective, this data suggests 120 min of match play causes deleterious effects on recovery and subsequent performance (in certain players), creating implications for the use of recovery modalities and training prescription following a match requiring ET. Practitioners should aim to identify players who may be particularly affected by 120 min of match play. With the inclusion of four substitutes instead of the traditional three being trialed and introduced in certain knockout tournaments, coaching staff can use this to substitute players whose performances and recoveries may be potentially compromised by an additional 30 min period.

## 5. Conclusions

Although we acknowledge the findings may reflect only the particular players involved, we conclude that ET creates a large demand on players who play the full 120 min, creating negative corollaries of recovery. This may influence some aspects of subsequent match performance and further recovery however, this is not uniform for all players. Despite its limitations, this work provides novel findings regarding the influence of ET on recovery and subsequent match performance in professional soccer players, and is a primer for future, more expansive investigations.

## Figures and Tables

**Table 1:** Comparison of recovery and performance variables between match-day 1 (MD1) and match-day 2 (MD2; required extra-time (ET)). ES = effect size. CMJ = countermovement jump. HI = high-intensity. Mechanical load derived from the manufacturer (PlayerLoad^TM^; Catapult Innovations, Melbourne, Australia).

	MD1	MD2	Mean Difference (±90% CL)	Qualitative Inference	ES
**Recovery measures**					
Mood	3 ± 1	3 ± 1	0	N.A.	0
Fatigue	4 ± 1	5 ± 1	1 ± 1	Most Likely Harmful	2
Sleep	3 ± 1	3 ± 1	0	N.A.	0
Muscle Soreness	4 ± 1	5 ± 1	1 ± 1	Very Likely Harmful	0.9
Wellness Score	50 ± 6	44 ± 7	−6 ± 5	Most Likely Harmful	−0.8
CMJ Height (cm)	48.5 ± 2.3	45.8 ± 4.4	−2.7 ± 5.4	Very Likely Harmful	−0.6
**Performance measures relative to match duration**					
Distance covered (m·min^−1^)	104.0 ± 6.6	111.5 ± 7.3	7.5 ± 2.4	Most Likely Increased	1.1
HI Distance (m·min^−1^)	4.9 ± 1.4	6.6 ± 0.8	1.7 ± 1.2	Most Likely Increased	1.3
Accelerations per min	2.4 ± 0.4	3.0 ± 0.4	0.6 ± 0.9	Very Likely Increased	1.6
Decelerations per min	1.0 ± 0.2	1.4 ± 0.3	0.4 ± 0.3	Most Likely Increased	2.4
Mechanical Load per min (AU)	10.3 ± 1.4	11.3 ± 1.5	1.1 ± 0.2	Most Likely Increased	0.7
**Performance measures (absolute values)**					
Distance covered (km)	10.4 ± 0.6	15.4 ± 0.9	4.9 ± 0.7	Most Likely Increased	7.5
HI distance (m)	438 ± 122	791 ± 99	353 ± 100	Most Likely Increased	2.9
Number of accelerations	216 ± 32	358 ± 52	139 ± 96	Most Likely Increased	4.4
Number of decelerations	89 ± 16	169 ± 38	80 ± 37	Most Likely Increased	5.1
Mechanical Load (AU)	922 ± 128	1357 ± 181	430 ± 70	Most Likely Increased	3.4
Pass accuracy (%)	88 ± 4	87 ± 8	N.A.	N.A.	0.3
Dribble accuracy (%)	70 ± 12	78 ± 12	N.A.	N.A.	0.7

**Table 2:** Comparison of recovery and performance variables between match-day 1 (MD1) and match-day 3 (MD3). CMJ = countermovement jump. HI = high-intensity. ES = effect size.

	MD1	MD3	Mean Difference (±90% CL)	Qualitative Inference	ES
**Recovery Measures**					
Mood	3 ± 1	3 ± 1	0	N.A.	0
Fatigue	4 ± 1	4 ± 1	0	N.A.	0
Sleep	3 ± 1	3 ± 1	0	N.A.	0
Muscle Soreness	4 ± 1	5 ± 1	1 ± 1	Very Likely Harmful	−0.5
Wellness Score	50 ± 6	46 ± 9	−4 ± 4	Very Likely Harmful	0.7
CMJ Height (cm)	48.5 ± 2.3	45.9 ± 5.1	−2.6 ± 4.8	Very Likely Harmful	1.1
**Performance measures relative to match duration**					
Distance covered (m·min^−1^)	104.0 ± 6.6	99.5 ± 12.6	−4.6 ± 20	Unclear	0.7
HI Distance (m·min^−1^)	4.9 ± 1.4	4.2 ± 0.6	−0.6 ± 1.6	Possibly Decreased	0.5
Accelerations per min	2.4 ± 0.4	2.3 ± 0.4	−0.1 ± 0.9	Unclear	0.2
Decelerations per min	1.0 ± 0.2	1.1 ± 0.3	0.1 ± 0.2	Unclear	−0.4
Mechanical Load per min (AU)	10.3 ± 1.4	10.9 ± 2.7	0.7 ± 3.4	Unclear	−0.5
Pass accuracy (%)	88.4 ± 4.9	78.7 ± 13.9	N.A.	N.A.	2
Dribble accuracy (%)	81.0 ± 26.8	68.6 ± 36.2	N.A.	N.A.	0.5

**Table 3:** Individual responses in recovery and performance variables between match-day 1 (MD1) and match-day 2 (MD2). Mechanical load derived from the manufacturer (PlayerLoad^TM^; Catapult Innovations, Melbourne, Australia).

		Player 1	Player 2	Player 3	Player 4
**Recovery Measures**					
Mood	MD1	4	2	3	3
	MD2	4	3	2	3
Fatigue	MD1	4	3	4	4
	MD2	5	4	5	5
Sleep	MD1	4	3	2	3
	MD2	4	2	3	3
Muscle Soreness	MD1	4	4	5	4
	MD2	5	4	6	5
Wellness Score	MD1	43	57	50	50
	MD2	36	54	43	43
CMJ Height (cm)	MD1	50.7	50.3	46.5	46.6
	MD2	43.0	50.0	41.2	49.0
**Performance measures relative to match duration**					
Distance covered (m·min^−1^)	MD1	98.6	98.1	111.0	108.3
	MD2	120.2	120.0	133.5	135.5
HI Distance (m·min^−1^)	MD1	3.3	4.9	4.7	6.6
	MD2	6.5	5.9	6.2	7.8
Accelerations per min	MD1	2.6	2.7	1.9	2.5
	MD2	2.9	2.4	3.5	3.2
Decelerations per min	MD1	0.8	0.9	1.0	1.2
	MD2	1.4	1.0	1.6	1.7
Mechanical Load per min (AU)	MD1	9.8	8.6	12.0	10.6
	MD2	11.1	9.4	13.0	11.8

**Table 4:** Individual responses in recovery and performance variables between match-day 1 (MD1) and match-day 3 (MD3). Mechanical load derived from the manufacturer (PlayerLoad^TM^; Catapult Innovations, Melbourne, Australia).

		Player 1	Player 2	Player 3	Player 4
**Recovery Measures**					
Mood	MD1	4	2	3	3
	MD3	4	2	3	4
Fatigue	MD1	4	3	4	4
	MD3	4	3	4	5
Sleep	MD1	4	3	2	3
	MD3	4	3	2	3
Muscle Soreness	MD1	4	4	5	4
	MD3	5	4	5	5
Wellness Score	MD1	43	57	50	50
	MD3	49	57	50	39
CMJ Height (cm)	MD1	50.7	50.3	46.5	46.6
	MD3	46.2	51.5	39.0	46.9
**Performance measures relative to match duration**					
Distance covered (m·min^−1^)	MD1	98.6	98.1	111.0	108.3
	MD3	105.4	101.2	81.3	109.9
HI Distance (m·min^−1^)	MD1	3.3	4.9	4.7	6.6
	MD3	4.0	3.5	5.0	4.4
Accelerations per min	MD1	2.6	2.7	1.9	2.5
	MD3	2	1.9	2.8	2.6
Decelerations per min	MD1	0.8	0.9	1.0	1.2
	MD3	0.9	0.8	1.3	1.2
Mechanical Load per min (AU)	MD1	9.8	8.6	12.0	10.6
	MD3	10.5	9.1	9.2	10.9
